# COVID-19 Vaccine Acceptance of Pregnant and Lactating Women (PLW) in Czechia: An Analytical Cross-Sectional Study

**DOI:** 10.3390/ijerph182413373

**Published:** 2021-12-19

**Authors:** Abanoub Riad, Anna Jouzová, Batuhan Üstün, Eliška Lagová, Lukáš Hruban, Petr Janků, Andrea Pokorná, Jitka Klugarová, Michal Koščík, Miloslav Klugar

**Affiliations:** 1 Department of Public Health, Faculty of Medicine, Masaryk University, Kamenice 5, 62500 Brno, Czech Republic; 394641@mail.muni.cz (E.L.); koscik@med.muni.cz (M.K.); 2 Czech National Centre for Evidence-Based Healthcare and Knowledge Translation (Cochrane Czech Republic, Czech EBHC: JBI Centre of Excellence, Masaryk University GRADE Centre), Faculty of Medicine, Institute of Biostatistics and Analyses, Masaryk University, Kamenice 5, 62500 Brno, Czech Republic; apokorna@med.muni.cz (A.P.); klugarova@med.muni.cz (J.K.); 3 Department of Obstetrics and Gynecology, University Hospital Brno and Medical Faculty, Masaryk University, Obilní Trh 11, 60200 Brno, Czech Republic; jouzova.anna@fnbrno.cz (A.J.); hruban.lukas@fnbrno.cz (L.H.); janku.petr@fnbrno.cz (P.J.); 4 Department of Gynecology and Obstetrics, Faculty of Medicine, Namık Kemal University, Namık Kemal Kampüs Caddesi No. 1, Merkez, Tekirdağ 59030, Turkey; bustun@nku.edu.tr; 5 Department of Health Sciences, Faculty of Medicine, Masaryk University, Kamenice 5, 62500 Brno, Czech Republic

**Keywords:** breastfeeding, COVID-19 vaccines, Czech Republic, decision making, health promotion, pregnant women, risk assessment

## Abstract

Pregnant and lactating women (PLW) represent a particular population subset with increased susceptibility for COVID-19 morbidity and mortality, even though the evidence about the safety and efficacy of COVID-19 vaccines was delayed due to their initial exclusion from development trials. This unclear situation could have led to increased COVID-19 vaccine hesitancy levels among PLW; therefore, this study aimed to evaluate the attitudes of Czech PLW towards COVID-19 vaccines and the determinants of their attitudes. An analytical cross-sectional survey-based study was carried out in the University Hospital Brno (South Moravia, Czechia) between August and October 2021. The study utilised a self-administered questionnaire (SAQ) adapted from previous instruments used for the same purpose. The SAQ included closed-ended items covering demographic characteristics, clinical and obstetric characteristics, attitudes towards COVID-19 vaccination, and potential psychosocial predictors of vaccine acceptance. Out of the 362 included participants, 278 were pregnant (PW) and 84 were lactating women (LW). The overall COVID-19 vaccine acceptance (immediate and delayed) level was substantially high (70.2%), with a significant difference between PW (76.6%) and LW (48.8%). Out of the 70.2% who agreed to receive the vaccine, 3.6% indicated immediate acceptance, and 66.6% indicated delayed acceptance. Only 13.3% of the participants indicated their acceptance of their physician’s vaccination recommendation during pregnancy or while lactating, and 62.2% were against it. Our results agreed with the recent studies that revealed that PW tended to have a high level of COVID-19 vaccine acceptance, and they were also inclined to resist professional recommendations because they predominantly preferred to delay their vaccination. The pregnancy trimester, education level, employment status, and previous live births were significant determinants for COVID-19 vaccine acceptance. The most commonly preferred vaccine type was mRNA-based vaccines, followed by viral vector-based and inactivated virus vaccines. The first top priority of PLW was vaccine safety for their children, followed by vaccine safety for the PLW and vaccine effectiveness. Regarding psychosocial predictors, media/social media, trust in the government, the pharmaceutical industry, and healthcare professionals, partners, and a positive risk-benefit ratio were significant promoters for COVID-19 vaccine acceptance. Findings from this study suggest that promotional interventions targeting PLW should use web platforms and focus on vaccine safety evidence, the expected benefits of vaccines and potential harms of the infection.

## 1. Introduction

The pregnancy and lactation periods are special times when susceptibility to morbidity and mortality for certain diseases increase [1]. Considering the COVID-19 disease, it has been shown that pregnant women (PW) are more likely to show symptoms, be hospitalised in intensive care units, and need ventilators compared to non-pregnant women (NPW) of the same age [2,3]. For this reason, it is of practical value that vaccination, which is the most effective method of coping with the current pandemic, is recommended for the population of pregnant and lactating women (PLW).

Vaccine hesitancy is one of the critical obstacles to the success of mass vaccination campaigns [4]. In the systematic review of Wilson et al., 2015, it was revealed that vaccine uptake during pregnancy is challenged by a wide array of barriers, including vaccine safety, perceived benefits, lack of recommendations by healthcare professionals, and lack of trust in healthcare providers and the pharmaceutical industry [5]. One can also put forward that making decisions during pregnancy leads to changes in the priorities, concerns and risk perception of PLW compared to other members of the society [6]. In this context, it is imperative to specifically investigate the factors that push this particular group to abstain from vaccination, thus achieving the success of vaccination programs [7].

Various studies have investigated the impact of media and social media on vaccine uptake, which indicated different directions of this impact, as media platforms may accelerate the infodemic during public health emergencies to a degree that hinders healthy decisions from being made [8,9,10]. Social media platforms can also be utilised to mitigate the risk of misinformation by providing high-quality information from authentic and engaging resources to PLW [11,12,13]. Confidence in government, the pharmaceutical industry, and health professionals has been consistently associated with vaccine acceptance [14,15]. Therefore, the World Health Organization (WHO) Strategic Advisory Group of Experts on Immunization (SAGE) suggested these predictors as part of the psychosocial elements of vaccine hesitancy questions compendium [16].

As of 1 November 2021, 6,246,557 individuals have received at least one dose of the COVID-19 vaccine in Czechia [17]. According to the State Institute for Drug Control (SÚKL) of the Czech Republic, a total of nine adverse reactions during pregnancy, puerperium, or the perinatal period were reported following COVID-19 vaccination [18]. Given the limitations of passive surveillance systems and their high odds of false-positive results, it is impossible to establish a causal relationship between COVID-19 vaccines and these events solely based on these reports [19]. COVID-19 vaccine acceptance among the Czech population has been variable and dependent on the population subgroup, impact of media and social media, trust in the pharmaceutical industry, knowledge, personal values, and fear of side effects [20,21,22]. However, PLW is a group that is traditionally a frequent target of vaccine hesitancy studies; there is still a lack of evidence on the prevalence of COVID-19 vaccine hesitancy among this particular population, especially in Central Europe [5,23]. Therefore, the aim of this study was to evaluate the attitudes of PLW in Czechia towards COVID-19 vaccination, as the primary objective was to estimate the prevalence of COVID-19 vaccine acceptance, while the secondary objective was to explore the potential determinants of COVID-19 vaccine acceptance.

## 2. Materials and Methods

### 2.1. Design

This analytical cross-sectional survey-based study was carried out between August and October 2021. We collected data from unvaccinated PLW to evaluate their COVID-19 vaccine-related attitudes. The study utilised a self-administered questionnaire (SAQ) that was designed and developed using KoBoToolbox (Harvard Humanitarian Initiative, Cambridge, MA, USA, 2021) [24]. The study was entirely designed and reported according to the Strengthening the Reporting of Observational Studies in Epidemiology (STROBE) guidelines for cross-sectional studies [25].

### 2.2. Participants

The target population of this study included both currently pregnant women (PW) and lactating women (LW). The study sample had been harvested from the outpatient gynecologic clinic of the University Hospital Brno (Fakultní nemocnice Brno “FN-Brno”), which is affiliated with the Department of Gynecology and Obstetrics at the Faculty of Medicine, Masaryk University [26].

The eligibility criteria encompassed: (a) any female patient in the reproductive age (15–44 years old) visiting the recruitment clinic during August–October 2021; (b) being currently pregnant at any trimester or having given a live birth within the last six months; (c) not having received any COVID-19 vaccine shot prior to filling in the questionnaire.

The target participants were primarily invited on-site to fill in the questionnaire while they were in the clinic’s waiting room, and hardcopy (printed) forms were used to collect their answers after they indicated their consent to join the study. Additionally, the study was promoted through the social channels of the University Hospital Brno (FN-Brno) to recruit further participants who filled in the questionnaire online using the digital form of KoBoToolbox.

**Figure 1:**
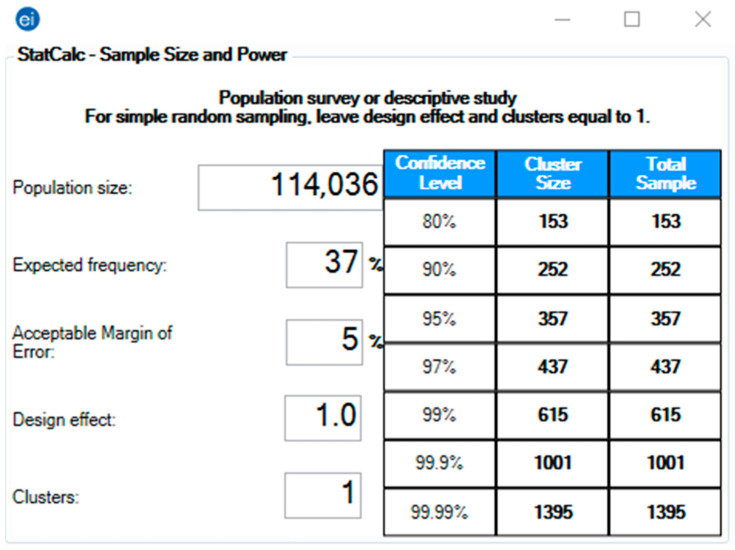
Sample size of Pregnant and Lactating Women (PLW) in the Czech Republic, Epi-Info version 7.2.4 [27,28,29,30,31].

The pragmatic sample size required for this study was 357, calculated using Epi-Info version 7.2.4 (CDC, Atlanta, GA, USA, 2020) [27]. The StatCalc module was used for population survey studies to achieve a 5% error margin and 95% confidence level [28]. The target population size was based on the number of live births in 2018 as reported by the Czech Statistical Office (CZSO), and the expected frequency (outcome probability) was assumed to be 37% based on the previous literature that reported vaccine acceptance among pregnant cohorts between 37% and 77% [29,30,31] (Figure 1).

**Figure 2:**
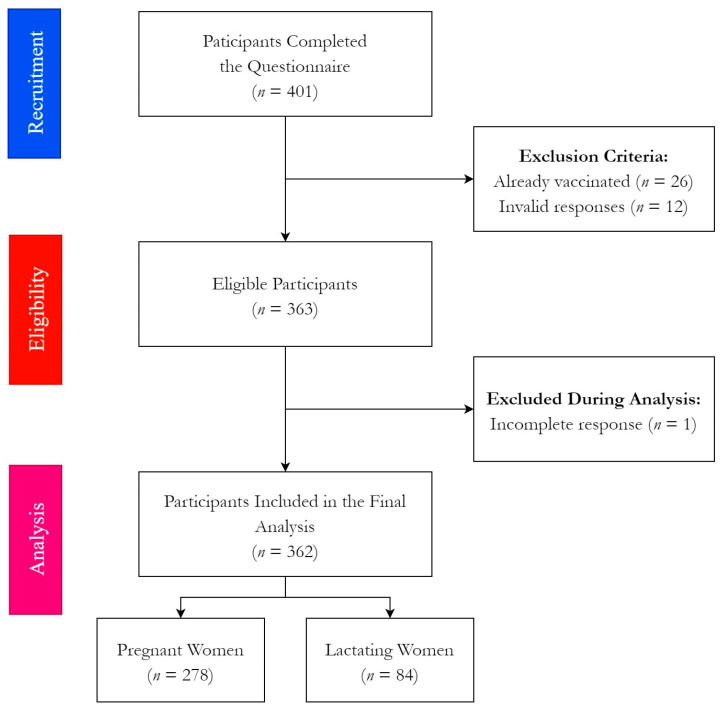
Flow chart of the participating pregnant and lactating women (PLW), University Hospital Brno, August–October 2021 (*n* = 362).

A total of 401 filled printed and digital forms were received by 25 October 2021. On eligibility verification, 38 responses were excluded due to being already vaccinated (*n* = 26) or containing invalid responses (*n* = 12). Prior to the statistical analysis phase, one more response was excluded due to incomplete answers. Finally, 362 responses were included in the downstream analysis (Figure 2).

### 2.3. Instrument

The SAQ used in this study consisted of 32 close-ended items. Most items were multiple-choice and Likert-scale questions, stratified in 4 categories and adopted from previous studies on vaccine hesitancy [14,16,20,30,31]. The first category was about demographic information, including current status (pregnant/new mother), age, education level, region, employment status, and household composition (children below four years, children and adolescents between 4 and 17 years, adults from 18 to 65 years, and seniors above 65 years). The second category was about the clinical anamnesis, including previous pregnancies, previous live births, complications and high-risk pregnancies, vaccination history during pregnancy and chronic illnesses. The third category was the COVID-19-related anamnesis, including previous infection, its onset (during pregnancy/before pregnancy), and its clinical course.

The fourth category included all items related to participants’ intentions towards COVID-19 vaccination and their psychosocial predictors. The psychosocial predictors of COVID-19 vaccine hesitancy were adapted from the compendium of vaccine hesitancy questions developed by the WHO-SAGE [16]. These predictors included the impact of media/social media, trust in government, trust in the pharmaceutical industry, trust in healthcare professionals, the influence of one’s spouse (partner), the perceived risk-benefit ratio, vaccine-related knowledge, and the vaccine’s safety.

In addition to the validated items of WHO-SAGE, the preferred vaccine type and the top priorities regarding the COVID-19 vaccines were surveyed. The intention towards COVID-19 vaccination was assessed by a multiple-choice question, “Do you intend to get vaccinated against COVID-19?” and the answers were classified into three groups “immediate acceptance”, “delayed or conditional acceptance”, and “rejection”. Moreover, the response to physicians’ recommendation of COVID-19 vaccination was evaluated on a 5-point Likert scale ranging from “definitely no” denoting 1, to “definitely yes” denoting 5.

The content validity of the used SAQ was assessed by a group of experts (*n* = 7) in gynaecology and obstetrics, public health, and health policy who provided their detailed feedback on the relevance, appropriateness, and clarity of the suggested items. Prior to data collection, the entire SAQ was revised in light of the provided feedback of the expert panel.

### 2.4. Ethics

The principles laid down by the Declaration of Helsinki guided the design and conduction of this study [32]. The Ethics Committee of the Faculty of Medicine, Masaryk University, reviewed the full protocol of this study and approved it on 19 May 2021 under the reference number Ref. 26/2021.

All participants were required to provide their informed consent digitally or by signature before joining the study, and no incentives or rewards were offered to increase the participation rate. The participants were allowed to leave the study any time before submitting the final form, and they were not obliged to explain their decision. This study data had been collected, processed, and stored per the General Data Protection Regulation (GDPR) of the European Union (EU) [33].

### 2.5. Analysis

The Statistical Package for the Social Sciences (SPSS) version 27.0 (SPSS Inc., Chicago, IL, USA, 2020) was used for both descriptive and inferential statistics of the study data [34]. The nominal variables, e.g., current status (pregnant/lactating), the dichotomous variables, e.g., employment status (yes/no), and the ordinal variables, e.g., education level (basic/secondary/bachelor’s/master’s), were described by frequencies (*n*) and percentages (%). The distribution properties of central tendency and dispersion, i.e., mean (µ), median, range and standard deviation (σ), were used to summarise the numerical variables such as the age, the number of previous pregnancies, and the psychosocial predictors as the answer “no” denoted 0, “not sure” denoted 1, and “yes” denoted 2.

Subsequently, Chi-squared test (χ^2^), Fisher’s exact test (for expected outcomes below 5), analysis of variance (ANOVA), and Mann–Whitney test (U) were used to evaluate the association of COVID-19 vaccine acceptance and response to physicians’ recommendations with demographic, anamnestic, and psychosocial risk factors. Logistic regression was used to estimate the adjusted odds ratio (AOR) of vaccine acceptance according to the proposed predictors. All inferential tests were performed with an assumed confidence level (CI) of 95% and significance level (Sig.) ≤ 0.05.

## 3. Results

### 3.1. Demographic Characteristics

Out of the 362 included participants, 278 were pregnant, and 84 were lactating women. The participants’ mean age was 31.48 ± 4.56 (19–44) years old, and their median age was 31 years old. Most pregnant women (PW) were in the third trimester (85.6%). The majority of participants held a master’s degree or higher (42.4%), followed by secondary (vocational) education (38.5%) and bachelor’s degree holders (14.4%). While 91.1% of the participants were employed prior to pregnancy, only 20.7% of the participants had been working in the healthcare sector (Table 1).

**Figure 3:**
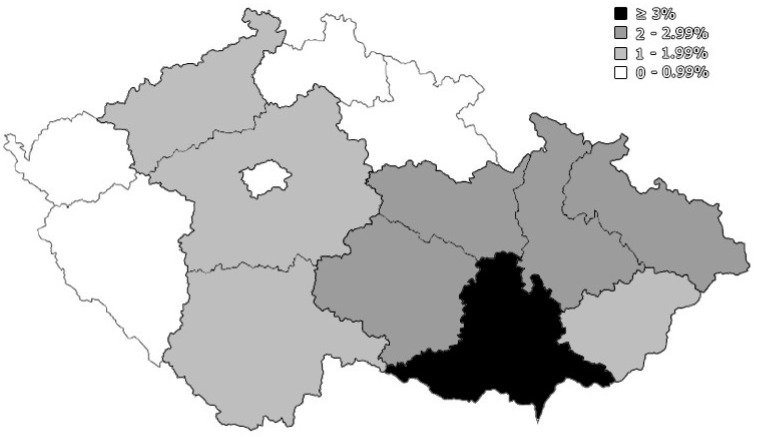
Geographic distribution of participating pregnant and lactating women (PLW), University Hospital Brno, August–October 2021 (*n* = 362).

The most represented region in this study was the South Moravian region (the home of FN-Brno), which constituted 80.9% of the total responses, followed by the neighbouring regions, i.e., Vysočina (2.8%), Olomouc (2.5%), and Pardubice (2.5%) (Figure 3).

Regarding the household composition of the participants, 49.9% of them had at least 1 child below 4 years old with a mean of 0.60 ± 0.68 children per participant, and 23% had at least 1 child or adolescent between 4 and 17 years old with a mean of 0.31 ± 0.62 adolescent per participant. About 97.2% of the participants had at least 1 adult member aged between 18 and 65 years old within their household, and only 7.8% of the participants had at least 1 senior above 65 years old, with a mean of 0.13 ± 0.62 seniors per participant.

### 3.2. Clinical and Obstetric Characteristics

On surveying their obstetric anamnesis, 59.9% of the participants had at least 1 previous pregnancy, with a mean number of 1 ± 1.04 previous pregnancies per participant. The majority of the previously pregnant participants had one live birth (57.2%), followed by two live births (23.6%), three live births (3.8%), and four live births (0.5%), and about 34.3% of the previous pregnancies had complications such as partial placental abruption, preterm birth, and subchorionic hematoma. High-risk pregnancies were reported by 19.1% of the participants, including gestational diabetes and advanced maternal age.

Less than half (45.3%) of the participants reported having at least 1 chronic illness, including allergy (21.3%), thyroid disease (12.7%), asthma (7.2%), skin-related disorders (6.4%), and anaemia (3.9%). Only 10 (2.8%) participants reported receiving vaccines during pregnancy other than the COVID-19 vaccine, including influenza and diphtheria, tetanus, and pertussis (DTP) vaccines (Table 2).

More than one fifth (21.5%) of the participants reported being previously infected by COVID-19, 37.2% of which were during their pregnancy. The clinical course of COVID-19 infection was classified as either mild (37.2%) or moderate (62.8%) according to the Australian guidelines for the care of people with COVID-19 [35] (Table 3).

### 3.3. COVID-19 Vaccine-Related Attitudes

When asked about their intention to receive the COVID-19 vaccine, two thirds of the participants (66.6%) indicated their acceptance; however, they preferred to wait a bit before receiving the vaccine, primarily until their pregnancy was successfully concluded or after weaning of their children. While only 3.6% of the participants indicated their immediate acceptance to receive the vaccine, up to 13.3% decided to accept the vaccine if their treating physicians advised them to receive the COVID-19 vaccine during pregnancy. In contrast to the 29.8% who exhibited no interest (rejected) to receive the COVID-19 vaccine under any condition, around 62.2% of the participants would decline the physicians’ vaccination recommendations (Table 4).

**Figure 4:**
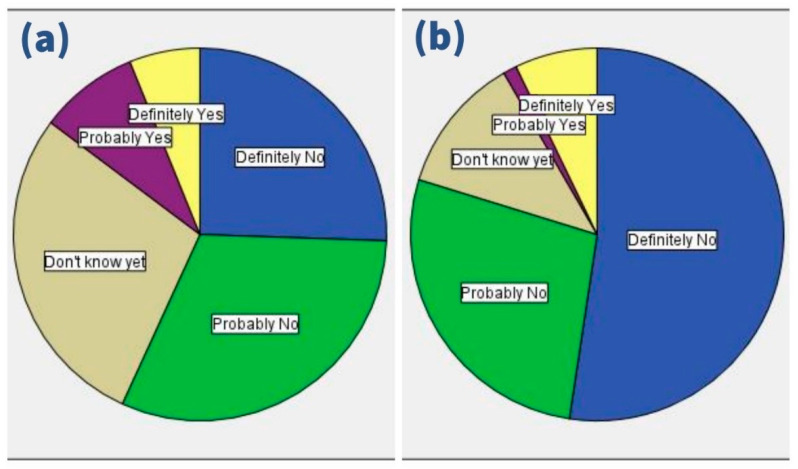
Response of (**a**) pregnant women and (**b**) lactating women to physician’s recommendation of COVID-19 vaccination, University Hospital Brno, August–October 2021 (*n* = 362).

On responding to the treating physicians’ recommendation, 14.7% of PW vs. 8.3% of LW would accept the COVID-19 vaccine, 28.4% vs. 11.9% would be hesitant, and 56.8% vs. 79.8% would reject the recommendation, respectively (Figure 4).

Less than one third (31.5%) of the participants had no preferred COVID-19 vaccine type, which corresponds with the percentage of the vaccine-resistant group (29.8%). The mRNA-based vaccines were the most preferred (58.6%), followed by the viral vector-based vaccines (6.6%) and the inactivated virus vaccines (3.3%).

In general, when the participants were asked about their first priority regarding the COVID-19 vaccine, safety for children (61.5%) was the most common answer. The most common second priority was safety for the mother (47%). The most common third and fourth priorities were the effectiveness for children’s immunisation, which were 35.2% vs. 41.4%, respectively (Table 5).

**Figure 5:**
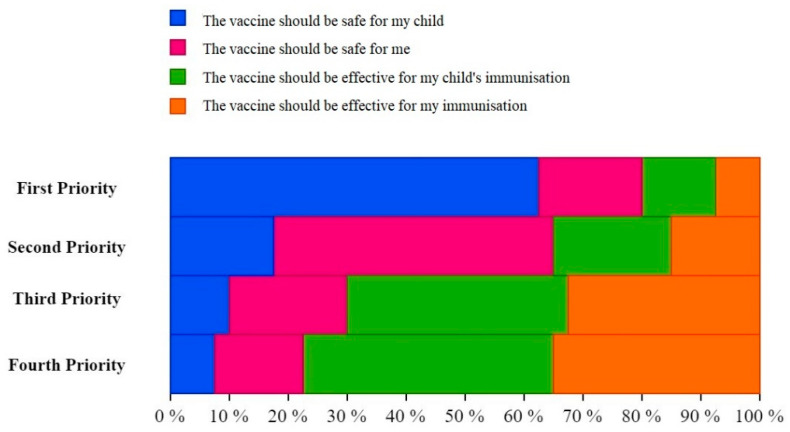
Top priorities of pregnant and lactating women (PLW) regarding COVID-19 vaccines, University Hospital Brno, August–October 2021 (*n* = 362).

There was no remarkable difference between PW and LW in the order of their priorities. For PW, the most common first priority was the safety for children (58.4%), the second priority was safety for the mother (46.2%), and the third and fourth priorities were the effectiveness for children’s immunisation at 33.6% and 38.3%, respectively. For LW, the most common first priority was the safety for children (71.1%), the second priority was safety for the mother (49.4%), and the third and fourth priorities were the effectiveness for children’s immunisation at 39.8% and 50.6%, respectively (Figure 5).

### 3.4. Psychosocial Predictors of Vaccine Hesitancy

The impact of media/social media on participants’ decisions was reported by only 8.3% of the participants. Most PLW (58.7%) did not think that the Czech government made decisions in the participants’ best interest concerning the offered vaccines.

Only 15% of the participants believed that the pharmaceutical companies were honest and transparent regarding safety data for pregnant women. Nearly one quarter (24.7%) agreed and another quarter (26.9%) disagreed with the notion that healthcare professionals would tell them honestly about the risks and benefits of vaccines, less than those (48.3%) who were still hesitant. The partners’ impact on vaccine decisions was also limited (10%), and up to half of the participants (50.6%) were unsure about the risk-benefit ratio of receiving COVID-19 vaccines.

Almost one-third of participants (32.5%) indicated that they had sufficient knowledge about the COVID-19 vaccines. The vast majority disagreed (82.4%) or were not sure (17.1%) that there was sufficient evidence suggesting the safety of COVID-19 vaccines for females, while only 2 (0.6%) participants thought there was enough evidence on COVID-19 vaccines’ safety (Table 6).

### 3.5. Analysis of COVID-19 Vaccine Acceptance

Pregnant women in their third trimester (80.7%) had a significantly (*χ*^2^ = 16.295; *Sig.* < 0.001) higher acceptance level of COVID-19 vaccine compared to the PW in the first trimester (41.7%).

Among the LW, there was no significant difference (*Sig.* = 0.095) in terms of age between the vaccine-accepting group (31.0 ± 4.7) and the vaccine-resistant group (32.8 ± 4.8). Additionally, there was no significant difference in terms of education level (*χ*^2^ = 1.512; *Sig*. = 0.670) or employment status (*χ*^2^ = 1.564; Sig. = 0.259). The lactating healthcare workers (72.2%) had a significantly higher (*χ*^2^ = 5.844; Sig. = 0.016) acceptance level compared to the non-healthcare workers (39.7%). The household composition had no significant association with the COVID-19 vaccine-related intentions of the LW. Similarly, the obstetric and COVID-19-related anamneses had no significant associations with the vaccination intentions. The psychosocial predictors were significantly higher (more positively answered) among the vaccine-accepting LW than the vaccine-resistant LW, except for knowledge and safety.

Among the PW, there was a significant difference (*Sig.* = 0.046) in terms of age between the vaccine-accepting group (31.6 ± 4.3) and the vaccine-resistant group (30.4 ± 5.0). Moreover, there was a significant difference in terms of education level (*χ*^2^ = 15.299; *Sig.* = 0.002) and employment status (*χ*^2^ = 9.856; *Sig.* = 0.002). The PW with a master’s degree or higher (85.3%) had a significantly higher acceptance level than the PW with basic education (42.9%). The pregnant healthcare workers (74%) had an insignificantly lower acceptance level compared to the non-healthcare workers (80.7%). The household composition had no significant association with the COVID-19 vaccine-related intentions of the PW, except for the presence of children and adolescents between 4 and 17 years old, which was associated with an increased rejection rate (38.9%). Similarly, the obstetric and COVID-19-related anamneses had no significant associations with the vaccination intentions, except for the item of previous live births. The PW with previous live births (26%) had a significantly (*χ*^2^ = 4.094; *Sig.* = 0.043) higher rejection level than the PW without previous live births (7.7%). Except for knowledge, all psychosocial predictors were significantly higher (more positively answered) among the vaccine-accepting PW than the vaccine-resistant PW (Table 7).

### 3.6. Analysis of Responses to Physicians’ Recommendations

The participants’ responses to the physicians’ recommendations for vaccination varied across several risk factors. The recommendation acceptance level was significantly higher (*χ*^2^ = 10.388; *Sig.* = 0.034) in the third trimester (16%) than the first trimester (8.3%). Age, employment status, and household composition had no significant association with the participants’ responses. The recommendation rejection level was the highest among PW with basic education compared to PW with higher education levels; contrarily, the recommendation rejection level was the lowest among LW with basic education compared to LW with higher education levels.

The obstetric and COVID-19-related anamneses had no significant association with the responses of PW, while the recommendation acceptance rate was significantly (Fisher’s exact test; *Sig.* = 0.009, 0.048, and 0.007) higher among LW without previous live births (40% vs. 2.2%), with complications (16.7% vs. 0%), and with chronic illnesses (15.8% vs. 2.2%) compared to their counterparts.

All psychosocial predictors were significantly higher (more positively answered) among the recommendation-accepting PW and LW compared to the recommendation-rejecting PW, except for media (LW) and knowledge (LW and PW) (Table 8).

### 3.7. COVID-19 Vaccine Acceptance among Pregnant Women

On running logistic regression for the demographic and anamnestic determinants of COVID-19 vaccine acceptance among PW, the third trimester had an AOR of vaccine acceptance which was 6.501 (CI 95%: 1.207–35.030) times more than the first trimester. The PW with a master’s degree or higher had an AOR of vaccine acceptance which was 5.992 (CI 95%: 1.116–32.164) times more than the PW with basic education. The employed PW and the PW without previous live births had AORs of vaccine acceptance which were 2.442 (CI 95%: 0.664–8.987) and 3.025 (CI 95%: 0.580–15.795) times more than their counterparts, respectively (Table 9).

For analysing the effect of each proposed psychosocial predictor of vaccine acceptance among PW, the regression analysis was adjusted for the significant demographic (trimester, education level, and employment status) and anamnestic (previous live births) risk factors. Trust in industry and healthcare professionals significantly increased the AORs of vaccine acceptance by 15.590 (CI 95%: 1.754–138.599) and 4.355 (CI 95%: 1.277–14.847) times more than their counterparts, respectively. Similarly, the PW with a favourable risk-benefit ratio had an AOR of vaccine acceptance which was 15.518 (CI 95%: 2.774–86.795) times more than their counterparts. Generally, all psychosocial predictors were associated with an increased AOR of vaccine acceptance, except for the perceived knowledge predictor, which was associated with a decreased AOR of 0.911 (CI 95%: 0.335–2.480) (Table 10).

## 4. Discussion

This study revealed that the overall COVID-19 vaccine acceptance level among Czech PLW was substantially high (70.2%), with a significant difference between PW (76.6%), who tended to be more positive towards vaccination, and LW (48.8%) who were inclined to resist vaccination. Out of the 254 (70.2%) participants who agreed to receive COVID-19 vaccination, 13 (3.6%) indicated immediate acceptance and 241 (66.6%) indicated delayed acceptance. Only 48 (13.3%) participants indicated their acceptance of the physician’s vaccination recommendation, and 225 (62.2%) were against the recommendation. Our results confirmed several findings presented by the previous studies on PLW intentions towards receiving COVID-19 vaccines. Tao et al., 2021 found that the COVID-19 vaccine acceptance level was 77.4% among Chinese PW in November 2020 [31]. In Turkey, Ayhan et al., 2021 found that the majority of PW (63%) rejected professionals’ recommendation of vaccination, which is similar to the level of recommendation-rejection among Czech PW (56.8%) [30].

The education level of Czech PW was a significant predictor for their vaccination intentions and their position on professionals’ recommendations; contrarily, Mohan et al., 2021 found the level of education was not a predictor for vaccine acceptance among perinatal women in Qatar [36]. While low education level was associated with increased COVID-19 vaccine acceptance odds among Chinese PW (AOR: 2.49; CI 95%: 1.13–5.51), high education level increased the vaccine acceptance odds among Czech PW (AOR: 5.99; CI 95%: 1.12–32.16) [31]. Besides COVID-19 vaccines, the education level of PW has been consistently reported as a positive predictor for childhood vaccine acceptance [37,38].

In our study, trimester was positively associated with the odds of COVID-19 vaccine acceptance, meaning that the third-trimester PW had the highest acceptance levels. One of the explanations for this finding is that an array of vaccines, including influenza (H1N1) and Tdap vaccines, are particularly recommended during the third trimester in multiple countries with various economic capacities; therefore, it can be assumed that vaccination during the third trimester is normalised and widely accepted [39,40,41]. One can also put forward the risk perception of medications, which makes PW avoid medicines during the first trimester, as it is depicted as a critical period for organogenesis [42,43]. As a confirmation, Blakeway et al., 2021 found within a sample of vaccinated PW in the UK that 85.7% of the vaccinees were in the third trimester versus 14.3% who were in the second trimester [44]. On the contrary, the first-trimester PW in Turkey had higher levels of COVID-19 vaccine acceptance compared to second- and third-trimester PW [30]. Suzuki 2020 concluded that anxiety and depressive symptoms were more frequently observed among the first trimester PW in Japan during the COVID-19 pandemic [45]. Moreover, Kenyan and Pakistani PW were more likely to accept maternal and influenza vaccines, respectively, during the first trimester compared to the subsequent trimesters [46,47].

Our employed PW (79.4%) had AORs of COVID-19 vaccine acceptance 2.44 (CI 95%: 0.66–8.99) times higher than the unemployed PW (52%). While employment status was not associated with COVID-19 vaccine acceptance among Chinese PW, Hoque et al., 2020 found that employed PW in South Africa had a higher odds ratio (OR: 4.21; CI 95%: 2.28–7.75) of COVID-19 vaccine acceptance compared to unemployed PW [31,48]. Moreover, Kalok et al., 2020 found that employed PW had a lower odds ratio (OR: 2.41; CI 95%: 1.50–3.86) of childhood vaccine hesitancy compared to unemployed PW in Malaysia [37]. However, maternal employment was not confirmed as a predictor for pregnancy outcomes; recent systematic reviews indicated that current employment and re-employment statuses were associated with better physical and mental health [49,50,51]. Additionally, employers may incentivise or even mandate vaccination as part of their employment conditions, especially in some sectors, including the healthcare sector; therefore, employed PW could be more open towards vaccination [52].

The household composition did not have an apparent influence on Czech PLW attitudes towards COVID-19 vaccines except for the presence of school-aged children, which was associated with vaccine resistance. In contrast, the number of school-aged children was positively correlated with the vaccine acceptance level among Turkish PW [30]. Likewise, the clinical and obstetric anamneses were not significantly associated with Czech PLW attitudes towards COVID-19 vaccination except for one element, which was the history of live births. The Czech PW with previous live births were more likely (AOR: 3.03; CI 95%: 0.58–15.80) to reject COVID-19 vaccines compared to the PW without previous live births. This difference can be attributed to perceived susceptibility, which refers to a “person’s subjective perception of the risk of acquiring an illness or disease” and increases the odds of adopting preventive health behaviour, as the PW with previous live births may feel less susceptible for adverse pregnancy outcomes [53,54,55].

When asked about their preferred COVID-19 vaccine type, 58.6% of Czech PLW disclosed their trust in mRNA-based vaccines, followed by viral-vector vaccines (6.6%) and inactivated virus vaccines (3.3%). The same order had been recently reported in Poland, as mRNA-based vaccines were the most trusted vaccines, followed by protein subunit, viral vector-based, inactivated virus and live attenuated vaccines [56]. Salerno et al., 2021 point out that Italian students were more hesitant (30.4%) and resistant (12.2%) regarding COVID-19 viral vector-based vaccines compared to mRNA-based vaccines (7.2% and 1%, respectively) [57]. Contrarily, a recent Chinese cross-sectional study revealed that 83.3% of the Chinese psychiatric patients preferred Chinese vaccines which are of inactivated virus, protein subunit, and viral vector-based types. In comparison, only 16.7% preferred the imported vaccines, including mRNA-based vaccines, thus reflecting the crisis of vaccine nationalism [58].

The first top priority for Czech PLW is the safety of COVID-19 vaccines for their unborn babies, which is consistent with previous studies. The multinational study of Skjefte et al., 2021 found that the top reason for COVID-19 vaccine reluctance among PW in 16 countries was the potentially harmful side effects for babies [7]. The second top priority for Czech PLW is the safety of COVID-19 vaccines for the mothers; this finding agrees with what had been reported earlier in China and Turkey among PW, where vaccine safety was a strong determinant of vaccine hesitancy [30,31]. However, the first-ever evidence regarding COVID-19 vaccine safety among PW was published in April 2021; prior to this date, there were thousands of vaccinated PW without specific safety signals detected by the national surveillance systems, e.g., the Vaccine Adverse Event Reporting System (VAERS) [59,60]. The limitation of COVID-19 vaccines’ safety evidence has resulted from the initial exclusion of PLW from the development trials; therefore, the obstetric societies have been calling upon manufacturers to include PLW in their vaccine trials in order to have reliable evidence on the potential/theoretical risks of vaccines, especially for fetuses and babies [61,62,63,64,65]. The post-marketing studies (phase IV) yielded no differences between pregnant and non-pregnant women regarding post-vaccination side effects’ prevalence or intensity [66,67,68]. Therefore, independent post-marketing studies of COVID-19 vaccines are believed to provide critical evidence to guide the public to make informed decisions [69,70,71,72,73,74,75,76].

The third top priority of the Czech PLW was the effectiveness of the COVID-19 vaccines for their fetuses/babies’ immune systems. Atyeo et al., 2021 designed a case-control study to investigate the placental transfer of antibodies against various viral infections and found that the placental transfer of SARS-CoV-2-specific antibodies was inefficient compared to pertussis-specific antibodies, especially during the third trimester [77]. The findings of Atyeo et al., 2021 have been confirmed by earlier studies that ruled out the vertical transmission of SARS-CoV-2 and found that the placental transmission of SARS-CoV-2-specific antibodies was inefficient [78]. Recently, emerging evidence has suggested that placental transfer of SARS-CoV-2-specific antibodies can be efficient to the degree that it will provide neonates with protection from COVID-19 [79,80]. PW with COVID-19 are more likely to be hospitalized and at an increased risk for ICU admission. There were justified concerns of vaccine side effects if administered during the gestational vulnerability window for any exposure, i.e., second and third trimesters of pregnancy [81]. Suppose the forthcoming evidence supports the hypothesis of effective placental transmission for SARS-CoV-2-specific antibodies. In that case, it will be of practical value to learn whether the transfer ratio differs according to gestational week/trimester, because the current body of evidence suggests that the second trimester might be the period of choice for COVID-19 vaccination [80]. Later evidence has indicated that COVID-19 mRNA-based vaccines generated robust humoral immunity in PLW, similar to that observed in non-pregnant women, and the response was significantly greater than from a natural infection. Immune transfer to neonates occurred via the placenta and breast milk with no observed harm to newborns [82].

The impact of media/social media on Czech PLW attitudes towards COVID-19 vaccination was significantly positive; therefore, local and national policymakers should act upon this finding by undertaking web-based interventions to boost vaccine uptake among Czech PLW [13]. In a three-arm randomised controlled trial, Glanz et al., 2017 concluded that PW who were exposed to a website with vaccine information and interactive social media components had infants with the highest level of childhood vaccine uptake compared to the other study groups, thus confirming the impact of web-based interventions targeting PW [11]. Additionally, web-based interventions that focused on third-trimester PW effectively increased maternal influenza vaccine uptake [12]. A recent qualitative study among skilled nursing staff warned against the misinformation that can be easily spread through social media against COVID-19 vaccines, thus mandating high-level interventions utilising web-based platforms to promote vaccines, especially among the high-risk groups, e.g., PLW [83]. Infertility and adverse pregnancy outcomes are the most common and sensitive topics of anti-vaccination campaigns that target PLW cohorts on social media platforms [8].

Trust in the government and the pharmaceutical industry were significant promoters of COVID-19 vaccine acceptance among Czech PLW. In the abovementioned multinational study of PW, trust in the political leadership was a significant promoter for COVID-19 vaccine among LW rather than PW [7]. French and Malaysian PW demonstrated the significant role of pharmaceutical industry trust as a promoter for vaccine acceptance [37,84]. Moreover, these two psychosocial predictors were consistently found to significantly impact COVID-19 vaccine acceptance among other populations, e.g., healthcare students globally, Austrian adults, British adults, Cameroonian adults, German healthcare workers, and French adults [85,86,87,88,89,90,91]. According to the German sociologist Ulrich Beck, vaccine hesitancy can be viewed as a manifestation of the threatening age of anxiety where Western populations lose trust in various institutions [92]. One of the implications of this narrative is that the pharmaceutical industry is framed as technocratic and trustworthy, while public suspicions of certain technologies should be perceived as ignorant and irrational [93]. Therefore, vaccine uptake research recommended focusing on educational campaigns to increase public knowledge of vaccination benefits and risks of diseases [94]. Contrarily, the promotional efforts among PLW were not similarly successful because they inadvertently individualised the perceived reproductive risk, which ought to be self-managed to secure the optimal benefits for their fetuses [6].

The degree of confidence in healthcare professionals to provide reliable and trustworthy information related to COVID-19 vaccine safety was a strong predictor (AOR: 4.36; CI 95%: 1.28–14.85) of vaccine acceptance among Czech PLW. This positive finding is consistent with the previous studies that concluded that PW are more likely to accept vaccines if they were recommended to them by trusted healthcare professionals and if they were accessible to them [5,95]. Previous studies have called for the education of healthcare professionals about maternal, antenatal, and childhood vaccination to enable professionals to provide high-quality and evidence-based recommendations regarding vaccines for hesitant PLW [96,97].

On the other hand, a substantial amount of Czech PLW (62.2%) declared their rejection of the immediate recommendation of their physicians to receive the COVID-19 vaccine. The first justification for this worrisome percentage is that COVID-19 vaccines’ safety evidence was relatively novel and probably not widespread yet. Second, PW may look for an active role in the decision-making process, especially if they experience high-risk pregnancies; therefore, they may resist the physicians’ recommendations if they are not based on reliable evidence [98]. Third, the evidence that suggested that the vaccination intentions of PW are affected by their trust in healthcare professionals predominantly originated from low-income settings; therefore, the situation may be different in Western societies due to libertarian and autonomy arguments [23].

The role of partners was positive in promoting COVID-19 vaccine acceptance among Czech PLW (AOR: 5.43; CI 95%: 0.57–52.01). Fabry et al., 2011 concluded that one of the H1N1 vaccine promoters among Canadian PW was advice from spouses [99]. In the Ivory Coast and Pakistan, the husband’s consent was essentially required for PW to receive tetanus vaccines during pregnancy due to the traditional role of the male as the head of the family and the primary decision maker [100,101]. The decision of Turkish PW regarding receiving the influenza vaccine during pregnancy had been made independently, without the involvement of spouses, in 37.9% of cases, after consultation with spouses in 51.5% of cases, and in 10.5% of cases, male spouses were the sole decision-makers [102]. Meharry et al., 2013 warned of the negative role that husbands/male spouses may play in PW vaccine acceptance due to their lack of knowledge or lower vaccine uptake [103].

As of 2019, about 582,336 foreign-born nationals were living in Czechia, representing around 5.1% of the total population in the Republic. There are also around a quarter-million individuals of the Indo-Aryan ethnicity known as “Romani” among the Czech nationals. While previous evidence has suggested that vaccine uptake of PW of ethnic minorities could be significantly higher, recent studies of COVID-19 vaccines uptake indicated the opposite and suggested ethnic minorities might be disadvantaged when it comes to COVID-19 vaccine campaigns [97,104]. One of the limitations of our study is that the instrument was exclusively available in the Czech language, and it did not ask about the ethnicity/origin of the participants.

Knowledge related to COVID-19 vaccines was the only psychosocial predictor in this study that did not promote vaccine acceptance among Czech PLW. According to the health belief model (HBM), knowledge interventions can target the constructs of perceived susceptibility, perceived severity, and perceived benefits [90]. Therefore, it can be assumed that higher vaccine-related knowledge should be associated with increased odds of vaccine acceptance. This unexpected finding should be interpreted carefully because the knowledge construct assessed in our study was the perceived knowledge, not the factual knowledge, and hence the participants may have overestimated their knowledge regarding COVID-19 vaccines. Future research on PLW should discriminate clearly between perceived knowledge and factual knowledge and test, if possible, the association between them in order to identify any discrepancies, if present. The illusion of knowledge is a challenge for health promotion interventions because it emerges due to discrepancies between perceived knowledge and factual knowledge [105]. This hypothesis can be confirmed because only 2 (0.6%) participants were aware about the currently existing evidence on COVID-19 vaccines safety for PW.

The risk-benefit ratio was a strong predictor of COVID-19 vaccine acceptance (AOR: 15.52; CI 95%: 2.77–86.80), representing a promising locus for public health interventions aiming to increase vaccine uptake by Czech PLW. According to the HBM theory, promotional interventions need to stress the perceived susceptibility of PW to COVID-19 infection that can lead to pregnancy complications, e.g., preterm birth, preterm premature rupture of the membranes, and maternal mortality in rare cases [106,107,108,109]. Likewise, the promotional interventions need to highlight the possible benefits of vaccination during pregnancy, including protection of prospective mothers from COVID-19 infection and potential immunologic benefits for the fetuses.

### 4.1. Strengths

To the best of the authors’ knowledge, this is the first study to explore vaccine acceptance and its determinants among PLW in Czechia, which represents central Europe’s particular cultural and economic region. This study was carried out after emerging preliminary evidence on COVID-19 vaccines’ safety to evaluate the immediate impact of this critical evidence. The study also included a large set of potential sociodemographic, clinical, and psychosocial determinants of COVD-19 vaccine acceptance; some of the parameters had been already validated by the WHO-SAGE. The preferred vaccine types and the position regarding physicians’ recommendations were explored for the first time among the PLW population. The anonymous nature of the questionnaire provided room for the participants to reflect their own opinions freely and trustworthily due to the minimisation of the Hawthorne bias.

### 4.2. Limitations

The first limitation of this study is the lack of ethnic minorities’ involvement; therefore, the findings of this study should be interpreted cautiously, as they only represent the Czech-speaking (native) population in Czechia. Second, the limited number of LW may have influenced the effect size of some risk factors, especially those found significant among PW [7]. Third, COVID-19 vaccine-related knowledge was assessed using a single item focusing on perceived knowledge; therefore, it was impossible to verify knowledge gaps among the surveyed population. Fourth, causality cannot be inferred from cross-sectional study designs, and self-reported outcomes may lead to misreporting, misclassification bias, and social desirability bias.

### 4.3. Implications

The findings of this study suggest that promotional interventions aiming to target PLW should emphasise the expected benefits of COVID-19 vaccines and the potential risks of COVID-19 infection during pregnancy. The present study also supports web-based interventions to increase PLW knowledge of COVID-19 vaccine safety and its expected benefits. Future studies of vaccine hesitancy among PLW need to clearly discriminate between perceived knowledge and factual knowledge to identify any potential knowledge gaps. Additionally, future research should include ethnic minorities and immigrant communities in Central European countries, as they could be affected differently by public health emergencies compared to native populations.

## 5. Conclusions

The overall COVID-19 vaccine acceptance (both immediate and delayed) level among Czech PLW was substantially high (70.2%), with a significant difference between PW (76.6%), who tended to be more positive towards vaccination, and LW (48.8%), who were inclined to resist vaccination. Out of the 70.2% who agreed to receive the vaccine, 3.6% indicated immediate acceptance, and 66.6% indicated delayed acceptance. Only 13.3% of the participants indicated their acceptance of the physician’s vaccination recommendation, and 62.2% were against the recommendation. Our results agreed with recent studies that revealed that PW tended to have high levels of COVID-19 vaccine acceptance, and they were also inclined to resist the professional recommendations because they predominantly preferred to delay their vaccination. The trimester, education level, employment status, and previous live births were significant determinants for COVID-19 vaccine acceptance among the target population. Regarding psychosocial predictors, media/social media, trust in the government, the pharmaceutical industry, and healthcare professionals, partners, and the risk-benefit ratio were significant promoters for COVID-19 vaccine acceptance. The level of COVID-19 vaccine-related knowledge could not predict vaccine acceptance, probably due to overestimating participants’ knowledge. Promotional interventions targeting PLW may use web platforms and focus on vaccine safety evidence, expected benefits of vaccines and potential harms of the infection.

## Figures and Tables

**Table 1:** Demographic characteristics of pregnant and lactating women (PLW), University Hospital Brno, August–October 2021 (*n* = 362).

Variable	Outcome	Frequency (*n*)	Percentage (%)
**Status**	Pregnant *	278	76.8%
	Lactating	84	23.2%
Trimester *	1st trimester (0–13 weeks)	12	4.3%
	2nd trimester (14–28 weeks)	28	10.1%
	3rd trimester (>28 weeks)	238	85.6%
**Age Group**	19–31 years-old	183	51.5%
	32–44 years-old	172	48.5%
**Education Level**	Basic (Elementary) Education	17	4.7%
	Secondary (Vocational) Education	139	38.5%
	Bachelor’s Degree	52	14.4%
	Master’s Degree or Higher	153	42.4%
**Employment Status**	Employed	329	91.1%
	Unemployed	32	8.9%
**Healthcare Profession**	Yes	68	20.7%
	No	260	79.3%
**Region**	South Moravian	292	80.9%
	Vysočina	10	2.8%
	Olomouc	9	2.5%
	Pardubice	9	2.5%
	Moravian-Silesian	8	2.2%
	Central Bohemian	7	1.9%
	Zlín	7	1.9%
	Other	19	5.3%
**Household** **Composition**	<4 years-old	178	49.9%
	4–17 years-old	82	23%
	18–65 years-old	349	97.2%
	>65 years-old	28	7.8%

* Pregnant women. Missing data of the age group was the heighest (1.9%), followed by household composition (0.8–1.4%), healthcare profession (0.6%), education level (0.3%), employment status (0.3%), and region (0.3%).

**Table 2:** Anamnestic characteristics of pregnant and lactating women (PLW), University Hospital Brno, August–October 2021 (*n* = 362).

Variable	Outcome	Frequency (*n*)	Percentage (%)
**Previous Pregnancies**	One	117	32.6%
	Two	59	16.4%
	Three	33	9.2%
	Four	6	1.7%
	None	144	40.1%
**Previous Live Births**	One	119	57.2%
	Two	49	23.6%
	Three	8	3.8%
	Four	1	0.5%
	None	31	14.9%
**Previous Pregnancies Complications**	Yes	72	34.3%
	No	138	65.7%
**Previous High-Risk Pregnancy**	Yes	69	19.1%
	No	293	80.9%
**Chronic Disease**	Allergy	77	21.3%
	Anaemia	14	3.9%
	Asthma	26	7.2%
	Cancer	0	0%
	Chronic Hypertension	3	0.8%
	Depression	3	0.8%
	Diabetes Mellitus	7	1.9%
	Gastrointestinal Disease	9	2.5%
	Obesity	12	3.3%
	Skin-related Disorder	23	6.4%
	Thyroid Disease	46	12.7%
	Total	164	45.3%
**Vaccine During** **Pregnancy**	Yes	10	2.8%
	No	352	97.2%

Missing data ranged between 0.3% to 0.8%.

**Table 3:** COVID-19-related anamnesis of pregnant and lactating women (PLW), University Hospital Brno, August–October 2021 (*n* = 362).

Variable	Outcome	Frequency (*n*)	Percentage (%)
**COVID-19 Infection**	Yes	78	21.5%
	No	284	78.5%
**Infection During Pregnancy**	Yes	29	37.2%
	No	49	62.8%
**Clinical Course**	Mild	29	37.2%
	Moderate	49	62.8%

No missing data.

**Table 4:** COVID-19 vaccine-related attitudes of pregnant and lactating women (PLW), University Hospital Brno, August –October 2021 (*n* = 362).

Variable	Outcome	Frequency (*n*)	Percentage (%)
**Intention**	Immediate Acceptance	13	3.6%
	Delayed Acceptance	241	66.6%
	Rejection	108	29.8%
**Position from Physician’s Recommendation**	Acceptance	48	13.3%
	Hesitancy	89	24.6%
	Rejection	225	62.2%

No missing data.

**Table 5:** Preferred vaccine type and top priorities of pregnant and lactating women (PLW), University Hospital Brno, August–October 2021 (*n* = 362).

Variable	Outcome	Frequency (*n*)	Percentage (%)
**Preferred Vaccine Type**	mRNA-based Vaccine	212	58.6%
	Viral Vector-based Vaccine	24	6.6%
	Inactivated Virus Vaccine	12	3.3%
	None	114	31.5%
**First Priority**	Safe for the child	209	61.5%
	Safe for the mother	63	18.5%
	Effective for the child	44	12.9%
	Effective for the mother	24	7.1%
**Second Priority**	Safe for the child	61	18.5%
	Safe for the mother	155	47%
	Effective for the child	61	18.5%
	Effective for the mother	53	16.1%
**Third Priority**	Safe for the child	36	10.9%
	Safe for the mother	68	20.6%
	Effective for the child	116	35.2%
	Effective for the mother	110	33.3%
**Fourth Priority**	Safe for the child	27	8.2%
	Safe for the mother	50	15.1%
	Effective for the child	137	41.4%
	Effective for the mother	117	35.3%

Missing data ranged between 6.1% to 8.8%.

**Table 6:** COVID-19 vaccine-related attitude predictors of pregnant and lactating women (PLW), University Hospital Brno, August–October 2021 (*n* = 362).

Variable	Outcome	Frequency (*n*)	Percentage (%)
**[Media]** Do reports you hear/read in the media/on social media make you re-consider the choice to take the COVID-19 vaccine?	No = 0	235	65.3%
	Not Sure = 1	95	26.4%
	Yes = 2	30	8.3%
**[Government]** Do you trust that your government is making decisions in your best interest concerning what vaccines are provided (e.g., your government purchases the highest quality vaccines available)?	No = 0	212	58.7%
	Not Sure = 1	118	32.7%
	Yes = 2	31	8.6%
**[Industry]** Do you trust pharmaceutical companies to provide credible data on COVID-19 vaccine safety and effectiveness?	No = 0	156	43.2%
	Not Sure = 1	151	41.8%
	Yes = 2	54	15%
**[Health Professional]** Do you trust your health care provider to tell you about the risks and benefits of vaccines honestly?	No = 0	97	26.9%
	Not Sure = 1	174	48.3%
	Yes = 2	89	24.7%
**[Partner]** My decision whether to get vaccinated or not is driven by my husband/partner?	No = 0	300	83.1%
	Not Sure = 1	25	6.9%
	Yes = 2	36	10%
**[Risk-benefit Ratio]** Do you think that the benefits of COVID-19 vaccines outweigh their reported side effects/adverse reactions?	No = 0	79	21.9%
	Not Sure = 1	182	50.6%
	Yes = 2	99	27.5%
**[Perceived Knowledge]** Do you feel you have enough information about COVID-19 vaccines and their safety?	No = 0	127	35.6%
	Not Sure = 1	114	31.9%
	Yes = 2	116	32.5%
**[Safety]** Do you think that there is enough evidence about COVID-19 vaccine safety during pregnancy?	No = 0	294	82.4%
	Not Sure = 1	61	17.1%
	Yes = 2	2	0.6%

Missing data ranged between 0.3% to 1.4%.

**Table 7:** Demographic, anamnestic and psychosocial risk factors of COVID-19 vaccine hesitancy among pregnant and lactating women (PLW), University Hospital Brno, August–October 2021 (*n* = 362).

Variable	Outcome	Lactating Women (*n* = 84)			Pregnant Women (*n* = 278)		
		Acceptance (*n* = 41)	Rejection (*n* = 43)	*Sig.*	Acceptance (*n* = 213)	Rejection (*n* = 65)	*Sig.*
**Trimester**	0–13 weeks	N/A	N/A	N/A	5 (41.7%)	7 (58.3%)	**0.008**
	14–28 weeks	N/A	N/A	N/A	16 (57.1%)	12 (42.9%)	**0.010**
	>28 weeks	N/A	N/A	N/A	192 (80.7%)	46 (19.3%)	**<0.001**
**Age**	*µ* ± *σ*	31.0 ± 4.7	32.8 ± 4.8	0.095	31.6 ± 4.3	30.4 ± 5.0	**0.046**
**Education** **Level**	Basic	2 (66.7%)	1 (33.3%)	0.611	6 (42.9%)	8 (57.1%)	**0.005**
	Secondary	14 (45.2%)	17 (54.8%)	0.609	81 (75%)	27 (25%)	0.611
	Bachelor’s	8 (61.5%)	5 (38.5%)	0.318	27 (69.2%)	12 (30.8%)	0.240
	Masters’/Higher	17 (45.9%)	20 (54.1%)	0.641	99 (85.3%)	17 (14.7%)	**0.004**
**Employment** **Status**	Yes	36 (46.8%)	41 (53.2%)	0.259	200 (79.4%)	52 (20.6%)	**0.002**
	No	5 (71.4%)	2 (28.6%)		13 (52%)	12 (48%)	
**Healthcare Professional**	Yes	13 (72.2%)	5 (27.8%)	**0.016**	37 (74%)	13 (26%)	0.295
	No	23 (39.7%)	35 (60.3%)		163 (80.7%)	39 (19.3%)	
**Household**	<4 years-old	39 (50.6%)	38 (49.4%)	1.000	74 (73.3%)	27 (26.7%)	0.250
	4–17 years-old	11 (39.3%)	17 (60.7%)	0.186	33 (61.1%)	21 (38.9%)	**0.003**
	18–65 years-old	39 (48.8%)	41 (51.2%)	0.494	206 (76.6%)	63 (23.4%)	1.000
	>65 years-old	5 (71.4%)	2 (28.6%)	0.432	14 (66.7%)	7 (33.3%)	0.282
**Previous Pregnancies**	Yes	25 (44.6%)	31 (55.4%)	0.212	122 (76.7%)	37 (23.3%)	0.970
	No	16 (59.3%)	11 (40.7%)		90 (76.9%)	27 (23.1%)	
**Previous** **Live Births**	Yes	19 (41.3%)	27 (58.7%)	0.162	97 (74%)	34 (26%)	**0.043**
	No	4 (80%)	2 (20%)		24 (92.3%)	2 (7.7%)	
**Pregnancies Complications**	Yes	9 (50%)	9 (50%)	0.621	46 (85.2%)	8 (14.8%)	0.080
	No	15 (42.9%)	20 (57.1%)		75 (72.8%)	28 (27.2%)	
**High-Risk Pregnancies**	Yes	10 (55.6%)	8 (44.4%)	0.518	40 (78.4%)	11 (21.6%)	0.735
	No	31 (47%)	35 (53%)		173 (76.2%)	54 (23.8%)	
**Chronic Illness**	Yes	17 (44.7%)	21 (55.3%)	0.497	97 (77%)	29 (23%)	0.896
	No	24 (52.2%)	22 (47.8%)		116 (76.3%)	36 (23.7%)	
**COVID-19**	Yes	7 (50%)	7 (50%)	0.922	50 (78.1%)	14 (21.9%)	0.746
**Infection**	No	34 (48.6%)	36 (51.4%)		163 (76.2%)	51 (23.8%)	
**During**	Yes	3 (75%)	1 (25%)	0.559	22 (88%)	3 (12%)	0.126
**Pregnancy**	No	4 (40%)	6 (60%)		28 (71.8%)	11 (28.2%)	
**Clinical**	Mild	3 (60%)	2 (40%)	1.000	16 (66.7%)	8 (33.3%)	0.086
**Course**	Moderate	4 (44.4%)	5 (55.6%)		34 (85%)	6 (15%)	
**Psychosocial** **Predictors**	Media	0.7 ± 0.9	0.2 ± 0.5	**0.001**	0.5 ± 0.6	0.2 ± 0.4	**<0.001**
	Government	0.6 ± 0.8	0.1 ± 0.5	**0.002**	0.7 ± 0.6	0.2 ± 0.5	**<0.001**
	Industry	0.9 ± 0.8	0.1 ± 0.4	**<0.001**	1.0 ± 0.6	0.2 ± 0.4	**<0.001**
	Health Professional	1.2 ± 0.8	0.4 ± 0.6	**<0.001**	1.1 ± 0.6	0.6 ± 0.7	**<0.001**
	Partner	0.3 ± 0.7	0.1 ± 0.4	**0.040**	0.3 ± 0.7	0.1 ± 0.4	**0028**
	Risk/Benefit Ratio	1.3 ± 0.7	0.6 ± 0.8	**<0.001**	1.2 ± 0.6	0.7 ± 0.6	**<0.001**
	Knowledge	1.0 ± 0.9	1.1 ± 0.9	0.562	0.9 ± 0.8	1.1 ± 0.9	0.153
	Safety	0.1 ± 0.3	0.1 ± 0.2	0.608	0.3 ± 0.5	0.1 ± 0.2	**<0.001**

Chi-squared test (*χ*^2^), Fisher’s exact test, analysis of variance test (ANOVA), and Mann–Whitney test (*U*) were used with a significance level (*Sig*.) ≤ 0.05.

**Table 8:** Demographic, anamnestic and psychosocial determinants of pregnant and lactating women (PLW)’s responses to physicians’ recommendations, University Hospital Brno, August–October 2021 (*n* = 362).

Variable	Outcome	Lactating Women (*n* = 84)				Pregnant Women (*n* = 278)			
		Rejection (*n* = 67)	Hesitancy (*n* = 10)	Acceptance (*n* = 7)	*Sig.*	Rejection (*n* = 158)	Hesitancy (*n* = 79)	Acceptance (*n* = 41)	*Sig.*
**Trimester**	0–13 weeks	N/A	N/A	N/A	N/A	10 (83.3%)	1 (8.3%)	1 (8.3%)	0.219
	14–28 weeks	N/A	N/A	N/A	N/A	22 (78.6%)	4 (14.3%)	2 (7.1%)	0.060
	>28 weeks	N/A	N/A	N/A	N/A	126 (52.9%)	74 (31.1%)	38 (16%)	**0.006**
**Age**	*µ* ± *σ*	32.2 ± 4.9	30.3 ± 5.1	31.3 ± 2.9	0.490	30.9 ± 4.6	32.0 ± 4.4	32.1 ± 4.0	0.100
**Education** **Level**	Basic	1 (33.3%)	2 (66.7%)	0 (0%)	**0.054**	10 (71.4%)	1 (7.1%)	3 (21.4%)	0.126
	Secondary	26 (83.9%)	4 (12.9%)	1 (3.2%)	0.506	70 (64.8%)	28 (25.9%)	10 (9.3%)	**0.052**
	Bachelor’s	9 (69.2%)	1 (7.7%)	3 (23.1%)	0.113	26 (66.7%)	6 (15.4%)	7 (17.9%)	0.150
	Masters’/Higher	31 (83.8%)	3 (8.1%)	3 (8.1%)	0.615	51 (44%)	44 (37.9%)	21 (18.1%)	**0.001**
**Employment** **Status**	Yes	63 (81.8%)	7 (9.1%)	7 (9.1%)	0.055	141 (56%)	74 (29.4%)	37 (14.7%)	0.616
	No	4 (57.1%)	3 (42.9%)	0 (0%)		16 (64%)	5 (20%)	4 (16%)	
**Healthcare Professional**	Yes	13 (72.2%)	2 (11.1%)	3 (16.7%)	0.459	33 (66%)	12 (24%)	5 (10%)	0.263
	No	49 (84.5%)	5 (8.6%)	4 (6.9%)		108 (53.5%)	62 (30.7%)	32 (15.8%)	
**Household**	<4 years-old	61 (79.2%)	9 (11.7%)	7 (9.1%)	0.697	62 (61.4%)	26 (25.7%)	13 (12.9%)	0.437
	4–17 years-old	25 (89.3%)	3 (10.7%)	0 (0%)	0.107	36 (66.7%)	10 (18.5%)	8 (14.8%)	0.164
	18–65 years-old	63 (78.8%)	10 (12.5%)	7 (8.8%)	1.000	154 (57.2%)	75 (27.9%)	40 (14.9%)	0.579
	>65 years-old	4 (57.1%)	2 (28.6%)	1 (14.3%)	0.198	13 (61.9%)	5 (23.8%)	3 (14.3%)	0.949
**Previous Pregnancies**	Yes	47 (83.9%)	6 (10.7%)	3 (5.4%)	0.242	91 (57.2%)	46 (28.9%)	22 (13.8%)	0.848
	No	19 (70.4%)	4 (14.8%)	4 (14.8%)		66 (56.4%)	32 (27.4%)	19 (16.2%)	
**Previous** **Live Births**	Yes	40 (87%)	5 (10.9%)	1 (2.2%)	**0.009**	78 (59.5%)	36 (27.5%)	17 (13%)	0.398
	No	2 (40%)	1 (20%)	2 (40%)		12 (46.2%)	10 (38.5%)	4 (15.4%)	
**Pregnancy Complications**	Yes	13 (72.2%)	2 (11.1%)	3 (16.7%)	**0.048**	28 (51.9%)	18 (33.3%)	8 (14.8%)	0.602
	No	31 (88.6%)	4 (11.4%)	0 (0%)		62 (60.2%)	28 (27.2%)	13 (12.6%)	
**High-Risk Pregnancies**	Yes	13 (72.2%)	2 (11.1%)	3 (16.7%)	0.379	28 (54.9%)	11 (21.6%)	12 (23.5%)	0.114
	No	54 (81.8%)	8 (12.1%)	4 (6.1%)		130 (57.3%)	68 (30%)	29 (12.8%)	
**Chronic Illness**	Yes	31 (81.6%)	1 (2.6%)	6 (15.8%)	**0.007**	70 (55.6%)	36 (28.6%)	20 (15.9%)	0.875
	No	36 (78.3%)	9 (19.6%)	1 (2.2%)		88 (57.9%)	43 (28.3%)	21 (13.8%)	
**COVID-19**	Yes	12 (85.7%)	1 (7.1%)	1 (7.1%)	1.000	33 (51.6%)	20 (31.3%)	11 (17.2%)	0.597
**Infection**	No	55 (78.6%)	9 (12.9%)	6 (8.6%)		125 (58.4%)	59 (27.6%)	30 (14%)	
**During**	Yes	3 (75%)	0 (0%)	1 (25%)	0.505	12 (48%)	6 (24%)	7 (28%)	0.166
**Pregnancy**	No	9 (90%)	1 (10%)	0 (0%)		21 (53.8%)	14 (35.9%)	4 (10.3%)	
**Clinical**	Mild	5 (100%)	0 (0%)	0 (0%)	1.000	17 (70.8%)	4 (16.7%)	3 (12.5%)	0.057
**Course**	Moderate	7 (77.8%)	1 (11.1%)	1 (11.1%)		16 (40%)	16 (40%)	8 (20%)	
**Psychosocial** **Predictors**	Media	0.4 ± 0.7	0.7 ± 0.9	0.6 ± 1.0	0.454	0.3 ± 0.6	0.6 ± 0.6	0.4 ± 0.6	**0.002**
	Government	0.3 ± 0.6	0.4 ± 0.7	1.3 ± 1.0	**0.003**	0.4 ± 0.6	0.7 ± 0.6	0.9 ± 0.6	**<0.001**
	Industry	0.3 ± 0.7	0.8 ± 0.8	1.4 ± 0.8	**<0.001**	0.5 ± 0.7	1.1 ± 0.6	1.2 ± 0.5	**<0.001**
	H. Professional	0.6 ± 0.8	1.0 ± 0.7	2.0 ± 0.0	**<0.001**	0.9 ± 0.7	1.3 ± 0.6	1.3 ± 0.5	**<0.001**
	Partner	0.1 ± 0.5	0.8 ± 0.9	0.3 ± 0.8	**<0.001**	0.2 ± 0.6	0.3 ± 0.6	0.5 ± 0.8	**0.020**
	Risk/Benefit Ratio	0.8 ± 0.8	1.2 ± 0.8	2.0 ± 0.0	**0.001**	1.0 ± 0.7	1.2 ± 0.6	1.3 ± 0.5	**0.003**
	Knowledge	1.0 ± 0.9	0.8 ± 0.9	1.6 ± 0.8	0.218	1.0 ± 0.8	0.8 ± 08	1.0 ± 0.7	0.085
	Safety	0.0 ± 0.2	0.2 ± 0.4	0.1 ± 0.4	**0.068**	0.1 ± 0.3	0.3 ± 0.5	0.6 ± 0.6	**<0.001**

Chi-squared test (*χ*^2^), Fisher’s exact test, analysis of variance test (ANOVA), and Mann–Whitney test (*U*) were used with a significance level (*Sig*.) ≤ 0.05.

**Table 9:** Analysis of COVID-19 vaccine acceptance among pregnant women, University Hospital Brno, August–October 2021 (*n* = 278).

Predictor	B (SE)	Wald	AOR (CI 95%)	*Sig.*
Second Trimester (vs. First Trimester)	0.163 (0.945)	0.030	1.177 (0.185–7.504)	0.863
Third Trimester (vs. First Trimester)	1.872 (0.859)	4.746	6.501 (1.207–35.030)	**0.029**
Secondary Education (vs. Basic Education)	1.299 (0.791)	2.697	3.665 (0.778–17.274)	0.101
Bachelor’s (vs. Basic Education)	1.026 (0.936)	1.201	2.790 (0.445–17.475)	0.273
Master’s or Higher (vs. Basic Education)	1.790 (0.857)	4.360	5.992 (1.116–32.164)	**0.037**
Employed (vs. Unemployed)	0.893 (0.665)	1.804	2.442 (0.664–8.987)	0.179
No Previous Live Births (vs. Previous Live Births)	1.107 (0.843)	1.724	3.025 (0.580–15.795)	0.189

Multivariate logistic regression of the COVID-19 vaccine acceptance (binary outcome) was performed with a significance level (*Sig*.) ≤ 0.05 and adjusted for trimester, education level, employment status, and history of live births.

**Table 10:** Psychosocial predictors of COVID-19 vaccine acceptance among pregnant women, University Hospital Brno, August–October 2021 (*n* = 278).

Predictor	B (SE)	Wald	AOR (CI 95%)	*Sig.*
Media: Yes (vs. No)	1.916 (1.183)	2.621	6.793 (0.668–69.087)	0.105
Government: Yes (vs. No)	0.888 (0.904)	0.966	2.431 (0.414–14.289)	0.326
Industry: Yes (vs. No)	2.747 (1.115)	6.070	15.590 (1.754–138.599)	**0.014**
Health Professional: Yes (vs. No)	1.471 (0.626)	5.527	4.355 (1.277–14.847)	**0.019**
Partner: Yes (vs. No)	1.692 (1.153)	2.156	5.433 (0.567–52.009)	0.142
Risk/Benefit Ratio: Yes (vs. No)	2.742 (0.878)	9.745	15.518 (2.774–86.795)	**0.002**
Knowledge: Yes (vs. No)	0.093 (0.511)	0.033	0.911 (0.335–2.480)	0.855
Safety: Yes (vs. No)	19.746 (40,192.969)	0.000	376 × 10^6^ (0.000–∞)	1.000

Multivariate logistic regression of COVID-19 vaccine acceptance (binary outcome) was performed with a significance level (*Sig*.) ≤ 0.05. The analysis was adjusted for the trimester, education level, employment status and previous live births.

## Data Availability

The data that support the findings of this study are available from the corresponding author upon reasonable request.
